# Supplementary material for: Modeling glioblastoma heterogeneity as a dynamic network of cell states
Source: Mol Syst Biol. 2021 Sep 16;17(9):e10105. doi: 10.15252/msb.202010105 (PMC8444284; doi:10.15252/msb.202010105)
Supplement: Supplementary file 6 — Source Data for Figure 5 [file MSB-17-e10105-s004.zip › Figure5A_sourcedata/GSEA_3071/hallmarks_stateIII.GseaPreranked.1623742540963/HALLMARK_INFLAMMATORY_RESPONSE.html]

Details for gene set HALLMARK\_INFLAMMATORY\_RESPONSE[GSEA]

|  || Dataset | state33071 |
| Phenotype | NoPhenotypeAvailable |
| Upregulated in class | na\_neg |
| GeneSet | HALLMARK\_INFLAMMATORY\_RESPONSE |
| Enrichment Score (ES) | -0.39723414 |
| Normalized Enrichment Score (NES) | -2.0278437 |
| Nominal p-value | 0.0011037528 |
| FDR q-value | 0.005750015 |
| FWER p-Value | 0.056 |
Table: GSEA Results Summary

  

Fig 1: Enrichment plot: HALLMARK\_INFLAMMATORY\_RESPONSE      
 Profile of the Running ES Score & Positions of GeneSet Members on the Rank Ordered List

  

| PROBE | GENE SYMBOL | GENE\_TITLE | RANK IN GENE LIST | RANK METRIC SCORE | RUNNING ES | CORE ENRICHMENT || 1 | HBEGF |  |  | 441 | -0.251 | -0.1011 | No |
| 2 | ICAM1 |  |  | 568 | -0.258 | -0.1207 | No |
| 3 | MYC |  |  | 599 | -0.260 | -0.1156 | No |
| 4 | TAPBP |  |  | 846 | -0.272 | -0.1654 | No |
| 5 | IL15 |  |  | 1334 | -0.297 | -0.2760 | No |
| 6 | BTG2 |  |  | 1350 | -0.297 | -0.2652 | No |
| 7 | KLF6 |  |  | 1596 | -0.312 | -0.3128 | No |
| 8 | PTGER4 |  |  | 1628 | -0.314 | -0.3052 | No |
| 9 | CDKN1A |  |  | 1631 | -0.314 | -0.2901 | No |
| 10 | ACVR1B |  |  | 1800 | -0.324 | -0.3172 | No |
| 11 | P2RX4 |  |  | 1861 | -0.328 | -0.3164 | No |
| 12 | PTPRE |  |  | 1880 | -0.328 | -0.3047 | No |
| 13 | KCNJ2 |  |  | 1974 | -0.334 | -0.3121 | No |
| 14 | TIMP1 |  |  | 2038 | -0.337 | -0.3116 | No |
| 15 | MXD1 |  |  | 2053 | -0.338 | -0.2984 | No |
| 16 | TLR3 |  |  | 2059 | -0.339 | -0.2829 | No |
| 17 | MET |  |  | 2138 | -0.344 | -0.2859 | No |
| 18 | PLAUR |  |  | 2181 | -0.347 | -0.2795 | No |
| 19 | IL7R |  |  | 2270 | -0.355 | -0.2846 | No |
| 20 | HAS2 |  |  | 2400 | -0.365 | -0.2997 | No |
| 21 | SGMS2 |  |  | 2450 | -0.369 | -0.2940 | No |
| 22 | MMP14 |  |  | 2811 | -0.400 | -0.3668 | No |
| 23 | PDE4B |  |  | 2878 | -0.406 | -0.3636 | No |
| 24 | LDLR |  |  | 2880 | -0.406 | -0.3437 | No |
| 25 | LIF |  |  | 2908 | -0.409 | -0.3304 | No |
| 26 | P2RX7 |  |  | 3016 | -0.422 | -0.3370 | No |
| 27 | ITGB8 |  |  | 3026 | -0.424 | -0.3183 | No |
| 28 | GABBR1 |  |  | 3226 | -0.447 | -0.3473 | No |
| 29 | ABCA1 |  |  | 3421 | -0.485 | -0.3732 | Yes |
| 30 | SERPINE1 |  |  | 3474 | -0.499 | -0.3618 | Yes |
| 31 | TNFSF10 |  |  | 3485 | -0.502 | -0.3395 | Yes |
| 32 | DCBLD2 |  |  | 3543 | -0.525 | -0.3281 | Yes |
| 33 | CD82 |  |  | 3569 | -0.534 | -0.3080 | Yes |
| 34 | ATP2B1 |  |  | 3580 | -0.539 | -0.2839 | Yes |
| 35 | RGS16 |  |  | 3616 | -0.555 | -0.2653 | Yes |
| 36 | CALCRL |  |  | 3631 | -0.565 | -0.2409 | Yes |
| 37 | TPBG |  |  | 3638 | -0.570 | -0.2141 | Yes |
| 38 | CSF1 |  |  | 3669 | -0.590 | -0.1926 | Yes |
| 39 | PDPN |  |  | 3676 | -0.597 | -0.1645 | Yes |
| 40 | PCDH7 |  |  | 3745 | -0.665 | -0.1490 | Yes |
| 41 | F3 |  |  | 3764 | -0.693 | -0.1193 | Yes |
| 42 | TNFAIP6 |  |  | 3780 | -0.713 | -0.0878 | Yes |
| 43 | INHBA |  |  | 3801 | -0.746 | -0.0559 | Yes |
| 44 | EDN1 |  |  | 3838 | -0.812 | -0.0249 | Yes |
| 45 | SLC1A2 |  |  | 3877 | -0.969 | 0.0134 | Yes |
Table: GSEA details [plain text format]

  

Fig 2: HALLMARK\_INFLAMMATORY\_RESPONSE: Random ES distribution      
 Gene set null distribution of ES for **HALLMARK\_INFLAMMATORY\_RESPONSE**
